# Supplementary figures and images for: Gene Coexpression Analysis Identifies Genes Associated with Chlorophyll Content and Relative Water Content in Pearl Millet
Source: Plants (Basel). 2023 Mar 22;12(6):1412. doi: 10.3390/plants12061412 (PMC10057621; doi:10.3390/plants12061412)

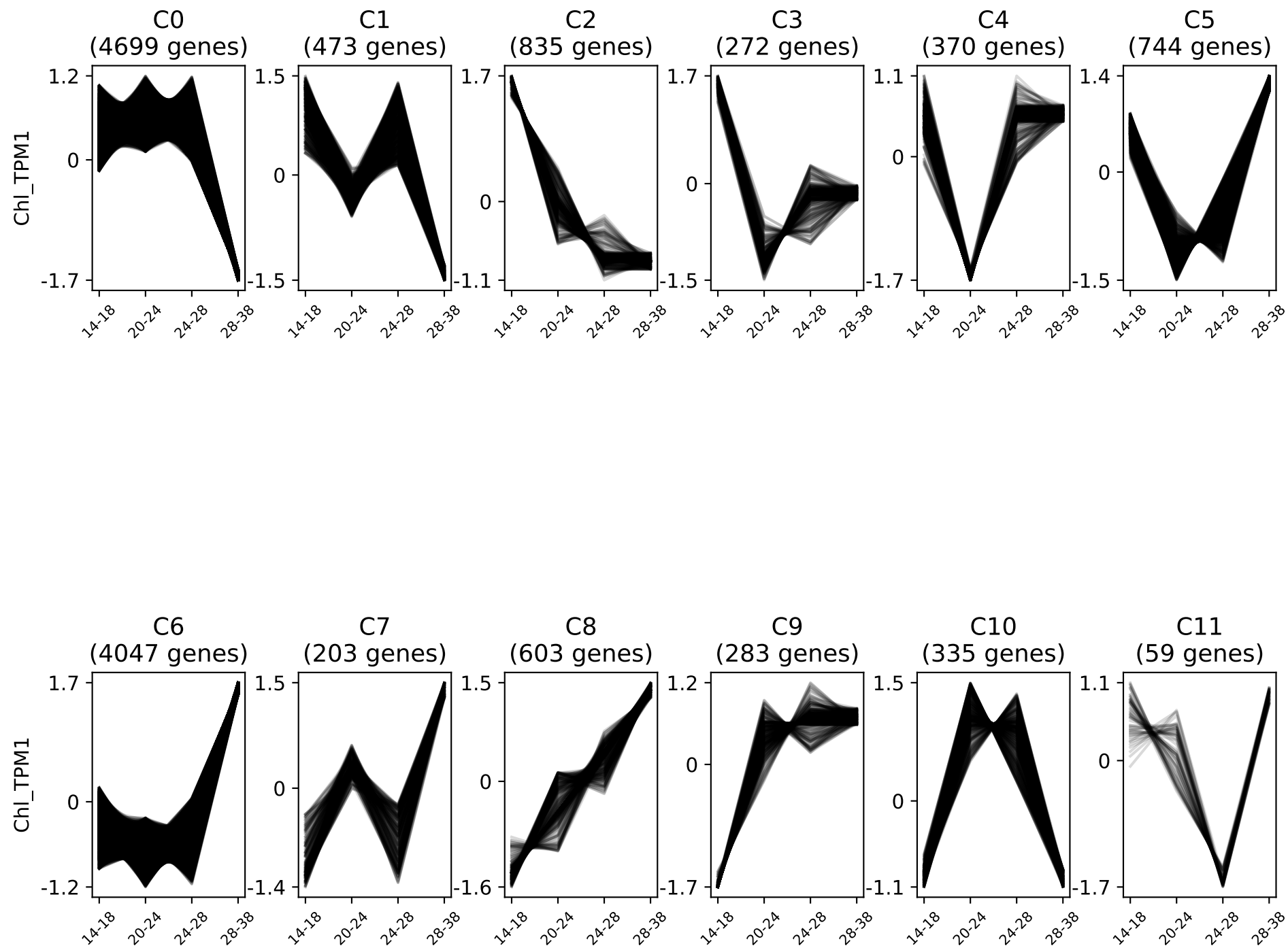

Supplement: Supplementary file 1 [file plants-12-01412-s001.zip › Supplementary Figure1.pdf]

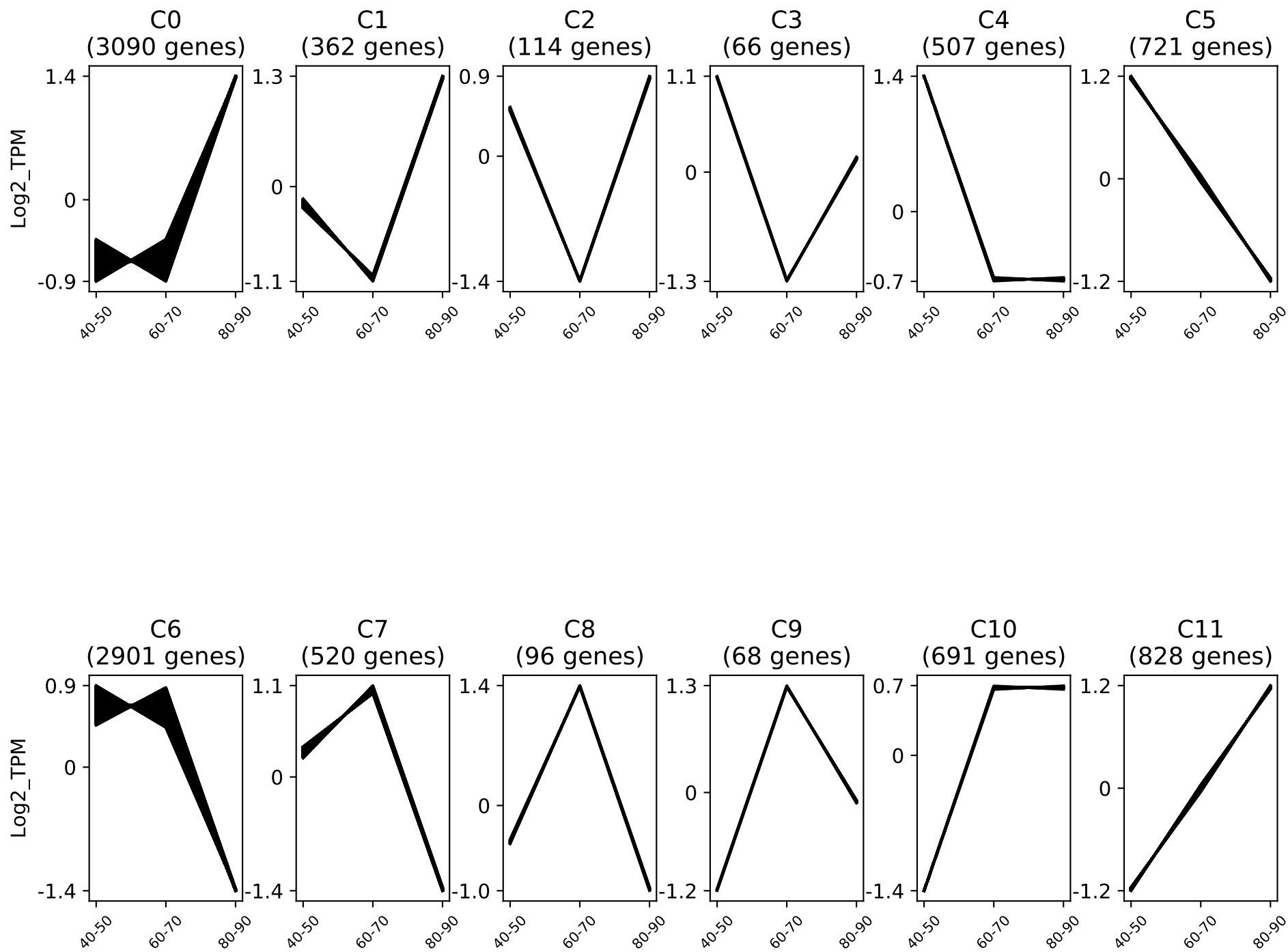

Supplement: Supplementary file 1 [file plants-12-01412-s001.zip › Supplementary Figure2.pdf]
